# Mendelian randomization and bioinformatics unveil potential links between gut microbial genera and colorectal cancer

**DOI:** 10.3389/fgene.2024.1379003

**Published:** 2024-11-21

**Authors:** Long Wu, Huan Wu, Fei Huang, Song Mu, Xiao-Yun Li, Bao-Fang Zhang, Yun-Huan Zhen, Hai-Yang Li

**Affiliations:** ^1^ Department of Anus and Intestinal Surgery, The Affiliated Hospital of Guizhou Medical University, Guiyang, China; ^2^ Department of Infectious Diseases, The Affiliated Hospital of Guizhou Medical University, Guiyang, China; ^3^ Department of Hepatobiliary Surgery, The Affiliated Hospital of Guizhou Medical University, Guiyang, China

**Keywords:** colorectal cancer, gut microbial genera, Mendelian randomization, potentiality, genes, single nucleotide polymorphisms

## Abstract

**Background:**

Colorectal cancer (CRC) poses a significant global health burden, with high incidence and mortality rates. Despite advances in diagnostic and therapeutic modalities, early diagnosis remains critical for improved outcomes. Recent research has realized the important role of gut microbiota in CRC development, highlighting the need to elucidate potential relationships.

**Methods:**

In this study, we employed Mendelian randomization (MR) to establish a robust potential link between gut microbial genera and CRC. Data from the MiBioGen database provided curated genome-wide association study (GWAS) summary datasets for microbial genera, while the Finngen database provided CRC outcome data. Instrumental variables (IVs) were identified based on genetic variants associated with gut microbiota. Various MR methods, including Inverse Variance Weighted (IVW), Weighted Median, Weighted Mode, Simple Mode, and MR-Egger, were employed to estimate potential effects. Functional analysis of genes near single nucleotide polymorphisms (SNPs) was performed to unravel potential pathways.

**Results:**

Analysis of microbial genera identified five potentially associated with CRC: *Eubacterium fissicatena group*, *Anaerofilum*, *Defluviitaleaceae UCG011*, *Ruminococcus 2*, and *Sutterella*. Notably, *Defluviitaleaceae UCG011* emerged as the only risk factor. Gene analysis revealed hub genes *PTPRD* and *DSCAM* near *Defluviitaleaceae UCG011* associated SNPs. Expression analysis showed that *PTPRD* decreased in colon cancer and *DSCAM* decreased in rectal cancer. The methylation status of the *PTPRD* gene promoter region indicated potential regulatory alterations.

**Conclusion:**

This study establishes a potential relationship between five specific gut microbial genera, particularly *Defluviitaleaceae UCG011*, and CRC. Hub genes *PTPRD* and *DSCAM* provide insights into potential molecular mechanisms, suggesting the potential role of *Defluviitaleaceae UCG011* in modulating the initiation and progression of CRC. Further research is essential to validate these associations and delve deeper into therapeutic implications.

## Introduction

Colorectal cancer (CRC) poses a significant global health challenge, with 1.8 million new diagnoses and 1 million deaths annually (Bray et al., 2018). Advances in diagnostic technologies and treatments, including surgery and chemotherapy, have led to an improved prognosis for CRC. Despite these advancements, CRC continues to be a global health issue, exerting a substantial impact on morbidity and mortality rates worldwide (Sung et al., 2021; Zheng RS. et al., 2023). The 5-year survival rate for early-stage (stages I and II) CRC patients can reach 80%, but it declines to less than 20% for those with advanced-stage disease, particularly stage IV (Biller and Schrag, 2021). Thus, identifying and understanding the factors and mechanisms that promote CRC development are crucial for improving treatment outcomes. CRC development is complex, involving a combination of genetic, environmental, and dietary factors (Thanikachalam and Khan, 2019). Recently, gut microbiota has been identified as playing a significant role in CRC development (Si et al., 2021).

The gut microbiota, an integral part of the human microbiome, is increasingly recognized as a key ecological factor influencing human health (Long et al., 2023). A substantial body of evidence indicates an association between gut microbiota and various digestive cancers, including colorectal, gastric, liver, and esophageal cancers, among others (Chen et al., 2022; Eun et al., 2014; Gao et al., 2023; Ni et al., 2022). Previous research suggests that the gut microbiota can influence CRC development by releasing various metabolites, proteins, and macromolecules that interact with the host’s colonic epithelium and immune cells (Avuthu and Guda, 2022). Some clinical evidence also underscores the role of the gut microbiota in modifying the therapeutic responses of patients with CRC to chemotherapy and immunotherapy (Wong and Yu, 2023). Moreover, the gut microbiota plays a crucial role in CRC metastasis, although the underlying mechanisms remain elusive (Zheng Z. et al., 2023). Studies have documented characteristic changes in gut microbiota across different CRC stages, with distinct alterations evident even at the colorectal adenoma stage (Dai et al., 2018; Liu et al., 2021; Wong and Yu, 2019). However, it is important to recognize that some observational studies may yield inconsistent conclusions due to limitations in assessing gut microbiota dysbiosis.

**FIGURE 1:**
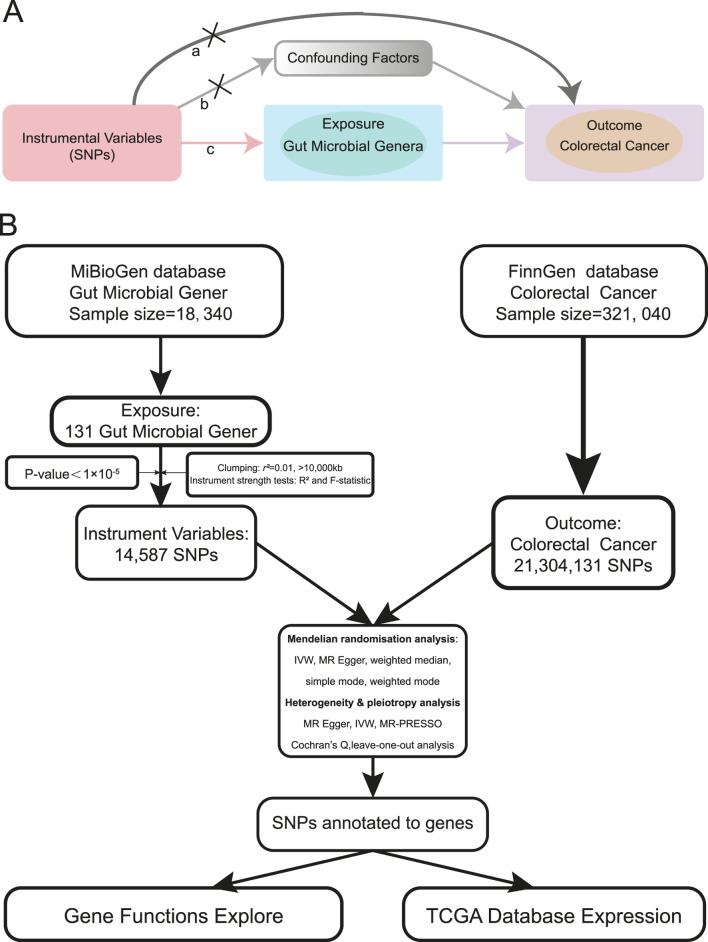
MR assumptions and analysis flow of this study. **(A)**. The MR assumptions (a. The exclusion restriction assumption; b. The independence assumption; c. The relevance assumption). **(B)**. The analysis flow of this study.

To establish a more robust potential relationship between gut microbial genera and CRC, we adopted Mendelian randomization (MR), a method that uses random genetic variants to mimic a randomized controlled trial (RCT), thereby circumventing environmental and lifestyle confounders. MR essentially functions as a natural RCT (Burgess et al., 2015; Larsson et al., 2023). MR is based on three key assumptions: 1) Genetic variations are associated with the risk factor of interest (relevance assumption); 2) Genetic variations are independent of confounders (independence assumption); and 3) Genetic variations affect the outcome only through the risk factor (exclusion restriction assumption) (Birney, 2022) (Figure 1A). Two-sample MR estimates potential effects by leveraging exposure and outcome data from different samples, thereby addressing the limitations of conventional observational studies (Davies et al., 2018; Lawlor, 2016).

In this study, we explored the potential relationship between gut microbial genera and CRC through a two-sample Mendelian randomization analysis. Additionally, we conducted a functional analysis of genes near single nucleotide polymorphisms (SNPs) identified in the study, to better understand the potential pathways through which these genera may influence CRC occurrence and development. Our aim was to elucidate the potential impact of gut bacterial genera on CRC, thereby providing new insights into the mechanisms underlying the development of colorectal cancer.

## Methods

### Data sources and screening of instrumental variables

We queried the MiBioGen database (https://mibiogen.gcc.rug.nl/) (Swertz et al., 2010; Swertz and Jansen, 2007; van der Velde et al., 2019) for curated GWAS summary datasets related to gut microbiota. Our focus was on GWAS data related to microbial genera. Using publicly accessible data, we conducted a two-sample MR study. We used genetic variants associated with gut microbial genera as instrumental variables (IVs), employing a *p*-value threshold of 1 × 10^-5^ based on previous reports (Bonder et al., 2016; Sanna et al., 2019). Additionally, we pruned the IVs based on linkage disequilibrium (LD) criteria (r^2^ ≥ 0.01, kb > 10,000) (Pritchard and Przeworski, 2001). Furthermore, we excluded SNPs that were palindromic with minor allele frequencies (Hemani et al., 2018a). After evaluating the IVs for exposure, we computed the F statistic for each individual variant. Each variant demonstrated an F statistic above 10, indicating a strong IV. It is generally accepted that an F statistic below 10 indicates a weak IV (Bowden et al., 2019; Burgess et al., 2011). GWAS data for colorectal cancer as the outcome was obtained from the most recent Finngen database (https://www.finngen.fi/) (Kurki et al., 2023). The FinnGen study is a large-scale genomics initiative that has analyzed over 500,000 Finnish biobank samples, correlating genetic variation with health data to understand disease mechanisms and predispositions (Kurki et al., 2022). This project is a collaboration between research organizations and biobanks in Finland and international industry partners. The latest release of the Finngen database is R10, updated in December 2023. The analysis flow of our study is illustrated in Figure 1B.

### Mendelian randomization analysis

This study employed the Inverse Variance Weighted (IVW) Random Effects as the primary MR method, complemented by four additional approaches: Weighted Median, Weighted Mode, Simple Mode, and MR-Egger. These diverse techniques provide a comprehensive perspective on the potential relationship, accounting for various assumptions and biases. After conducting a two-sample MR analysis on the overall microbial genera, those potentially associated with colorectal cancer were identified using the IVW method, with a significance threshold of *p* < 0.05. All *p*-values were adjusted using the Benjamini–Hochberg method to evaluate false discovery rate (FDR) (Benjamini and Hochberg, 2024). The results were visualized using forest plots and circos plots. Subsequently, a dedicated MR analysis was performed on the genera identified as potentially associated with colorectal cancer. All MR analyses were conducted using RStudio software (Version: 2023.06.0 Build 421) and R software (Version: 4.3.2) (Hemani et al., 2018b).

### Heterogeneity and pleiotropy test

We assessed heterogeneity among SNPs using Cochran’s Q-statistics (Egger et al., 1997) and the *I* ^2^ statistic (Bowden et al., 2016; Higgins and Thompson, 2002). We used MR-Egger and Mendelian Randomization Pleiotropy Residual Sum and Outlier (MR-PRESSO) to assess horizontal pleiotropy of SNPs(Verbanck et al., 2018). Additionally, we conducted a ‘Leave-one-out’ analysis to explore the influence of individual SNPs on the overall association (Mikshowsky et al., 2017).

### Gene functions explore

Based on the MR analysis results, we compiled a list of all SNPs included in the study. Using R software, we searched for genes within 50 base pairs of each SNP. We then explored the interactions among the identified genes using the STRING database (https://string-db.org/)(Szklarczyk et al., 2019) and Cytoscape software (Version 3.10.1) (Maere et al., 2005) to identify key functional genes. We performed functional enrichment analysis of these genes using Gene Ontology (GO) (Ashburner et al., 2000; Gene Ontology et al., 2023) and the Kyoto Encyclopedia of Genes and Genomes (KEGG) (Kanehisa and Goto, 2000), and visualized the results for better interpretation using RStudio software (Version: 2023.06.0 Build 421). Next, we analyzed the expression profiles of the relevant genes in colorectal cancer using The Cancer Genome Atlas (TCGA) database. We further analyzed the methylation status of the PTPRD gene promoter region using the TCGA database. We then analyzed the relationship between PTPRD gene expression and colon cancer survival by mapping the Kaplan-Meier (KM) survival curve.

## Results

### Research datasets and instrumental variables

We obtained GWAS data summaries for 131 microbial genera from the MiBioGen database (Kurilshikov et al., 2021). After filtering with a significance threshold of *p* < 1 × 10^-5^, we identified 14,587 SNPs. Subsequently, we pruned for linkage disequilibrium and selected strong instrumental variables, resulting in a final set of 1,531 eligible SNPs for inclusion in the MR analysis. Details and F statistics for the SNPs included in this study are provided in Supplementary Table S1. The outcome data were retrieved from the FinnGen database, focusing on the colorectal cancer phenotype. The GWAS data included in the MR analysis consisted of 6,847 cases and 314,193 controls, encompassing a total of 21,304,131 SNPs. Information on the GWAS data used in this study is presented in Table 1.

**TABLE 1:** Information of GWAS data used in this study.

Data type	Phenotype	Source	Website	Sample size	Year
Exposure	Gut Microbial Genera	the MiBioGen consortium	https://mibiogen.gcc.rug.nl/	18,340 (24 cohorts)	2021
Outcome	Colorectal Cancer	the FinnGen consortium	https://www.finngen.fi/	321,040 (6,847 cases; 314,193 controls)	2023

Abbreviations: GWAS, Genome-wide association study.

### Mendelian randomization results

**FIGURE 2:**
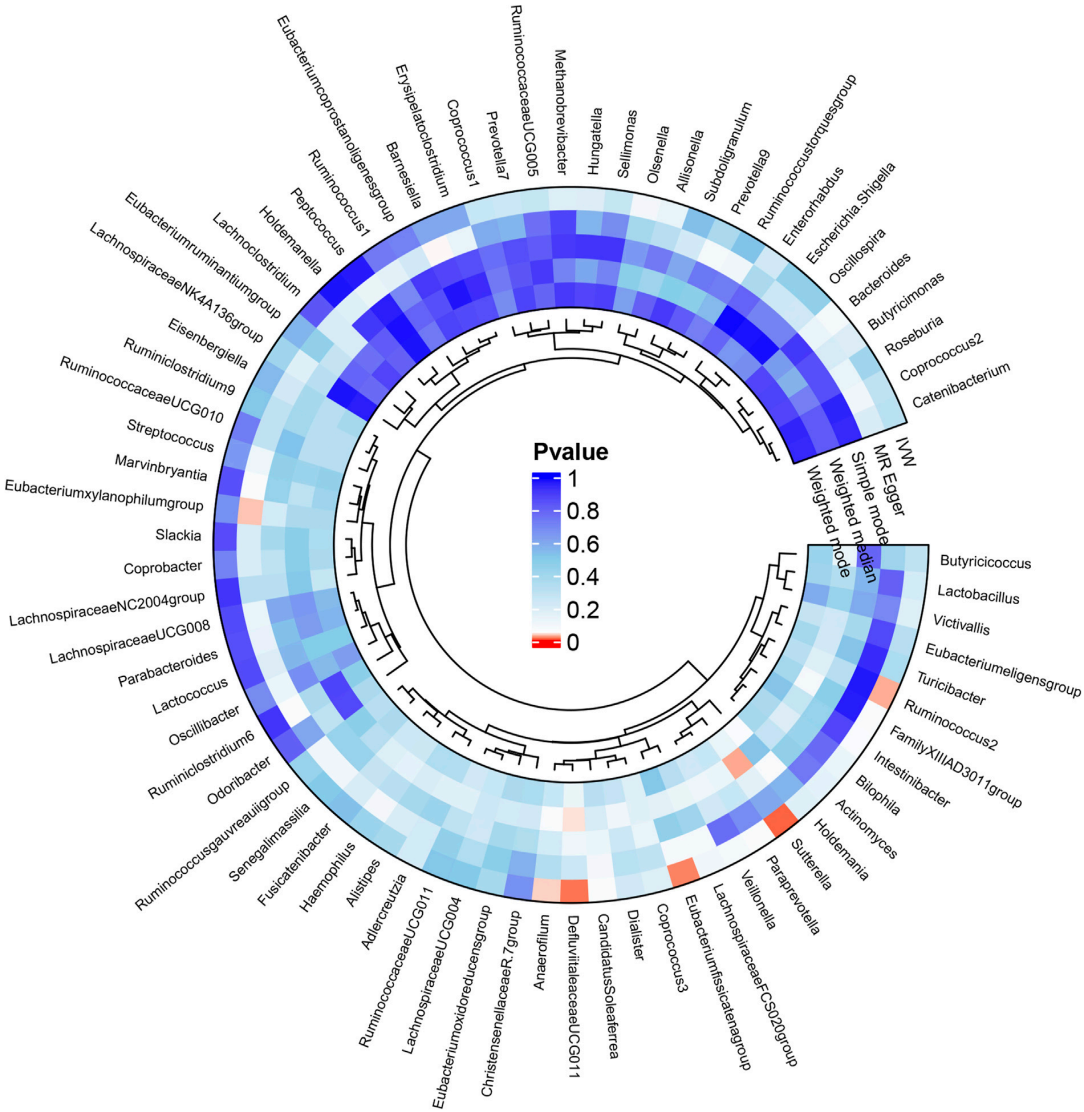
Circos plot of MR results for the associations between all gut microbial genera and the risk of colorectal cancer. From the inner to outer circles, they represent the estimates of weighted mode, weighted median, simple mode, MR-Egger, and inverse-variance weighted methods, respectively. The shades of color reflect the magnitude of the value of *p*-value, and red shades reflect *p*-value <0.05.

**FIGURE 3:**
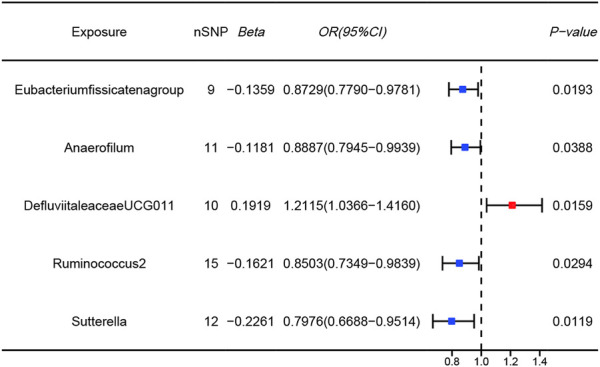
Forest plots of MR results for five gut microbial genera potentially associated with colorectal cancer using IVW method (Blue dots represent protective factors; Red dot represents risk factors).

**FIGURE 4:**
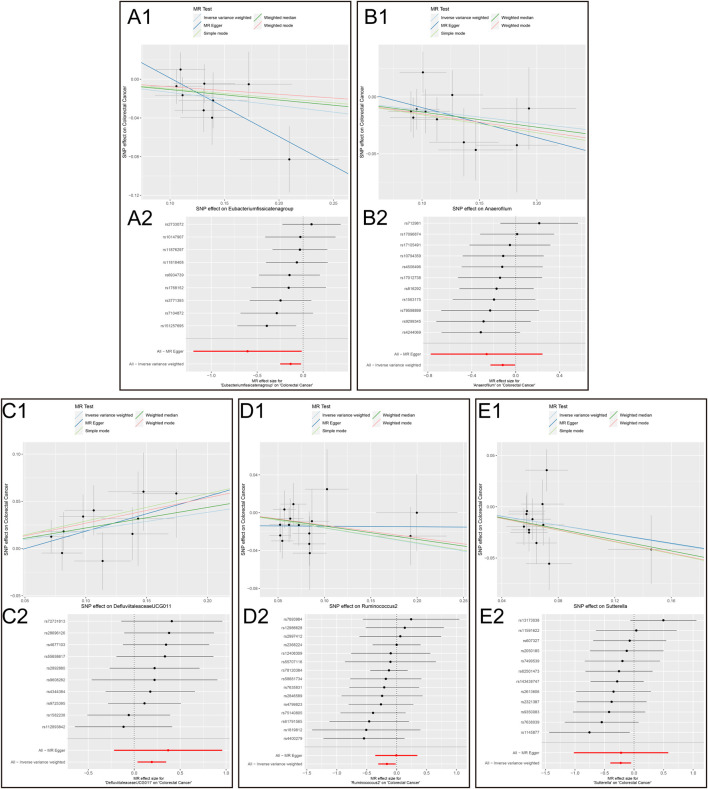
Scatter plots and MR effect size plots for five gut microbial genera potentially associated with colorectal cancer. **(A)** Scatter plots **(B)** MR effect size plots (1. *Eubacterium fissicatena group* on Colorectal Cancer. 2. *Anaerofilum* on Colorectal Cancer. 3. *Defluviitaleaceae UCG011* on Colorectal Cancer. 4. *Ruminococcus* 2 on Colorectal Cancer.5. *Sutterella* on Colorectal Cancer).

The results of the MR analysis for all microbial genera are provided in Supplementary Table S2. Among them, 75 genera in the IVW method were visually represented in a circos plot, with those potentially associated with colorectal cancer highlighted in red (Figure 2). As shown in Figure 2, five genera demonstrated a potential relationship with colorectal cancer, namely, *Eubacterium fissicatena group* [*Beta* = −0.136, *OR (95%CI)* = 0.873 (0.779–0.978), *p* = 0.019], *Anaerofilum* [*Beta* = −0.118, *OR (95%CI)* = 0.889 (0.795–0.994), *p* = 0.039], *Defluviitaleaceae UCG011* [*Beta* = 0.192, *OR (95%CI)* = 1.212 (1.037–1.416), *p* = 0.016], *Ruminococcus 2* [*Beta* = −0.162, *OR (95%CI)* = 0.850 (0.735–0.984), *p* = 0.029], and *Sutterella* [*Beta* = −0.226, *OR (95%CI)* = 0.798 (0.669–0.951), *p* = 0.012]. The MR results for these five genera were further illustrated using forest plots (Figure 3) and scatter plots (Figure 4). Additionally, *Eubacterium xylanophilum group* [*Beta* = −0.781, *OR (95%CI)* = 0.458 (0.247–0.850), *p* = 0.035]and *Barnesiella* [*Beta* = −0.902, *OR (95%CI)* = 0.406 (0.179–0.920), *p* = 0.049] showed a potential relationship with colorectal cancer in the MR Egger method, while *Defluviitaleaceae UCG011* [*Beta* = 0.218, *OR (95%CI)* = 1.243 (1.007–1.535), *p* = 0.043] and *Sutterella* [*Beta* = −0.269, *OR (95%CI)* = 0.764 (0.601–0.972), *p* = 0.028]exhibited a potential relationship in the Weighted Median method. We adjusted the *p*-values of IVW method and found that all FDR were close to 1 (Supplementary Table S3).

### Heterogeneity and pleiotropy test results

**FIGURE 5:**
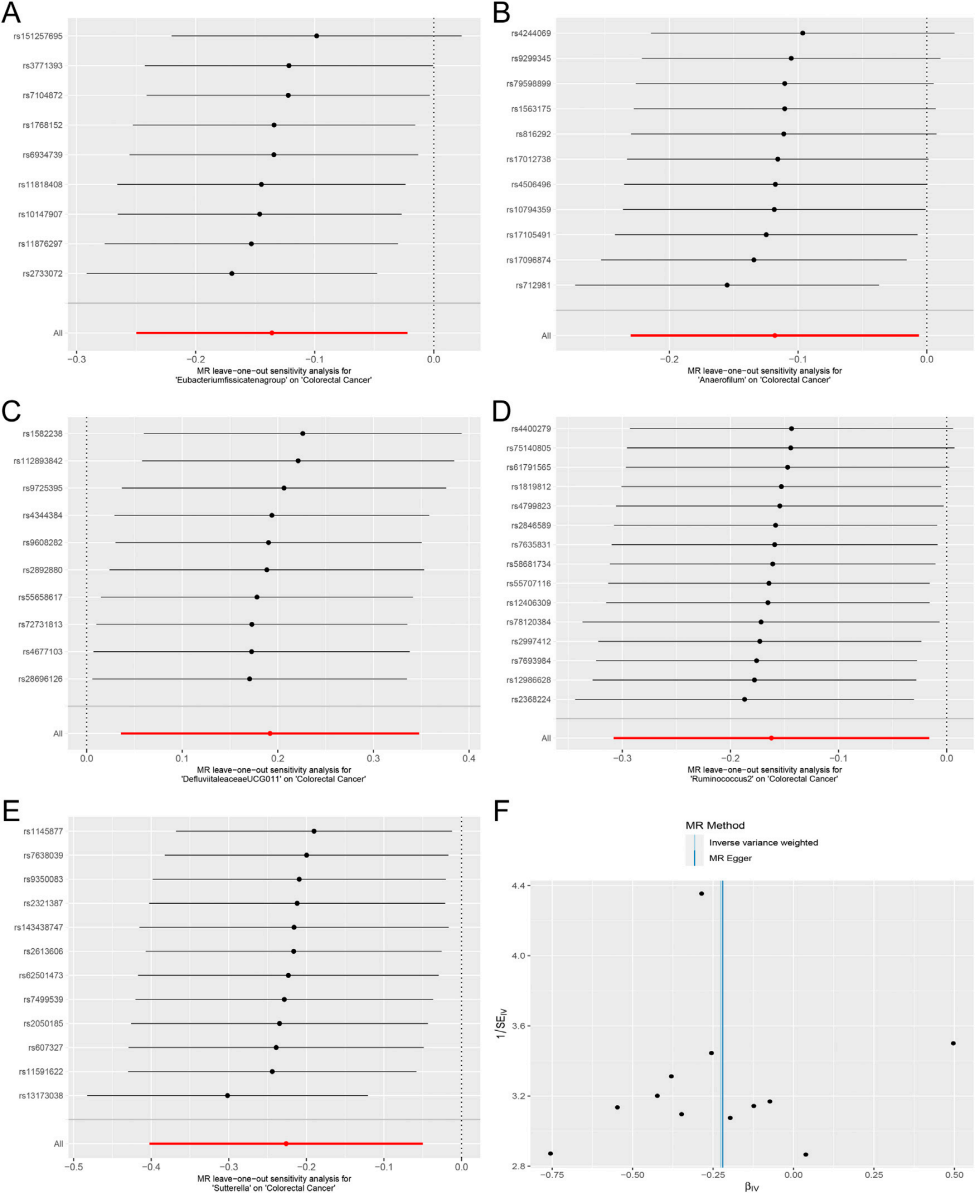
Forest plots of MR Leave-one-out sensitivity analysis and the funnel plot. **(A)**. MR Leave-one-out sensitivity analysis for *Eubacterium fissicatena group* on Colorectal Cancer. **(B)**. MR Leave-one-out sensitivity analysis for *Anaerofilum* on Colorectal Cancer. **(C)**. MR Leave-one-out sensitivity analysis for *Defluviitaleaceae UCG011* on Colorectal Cancer. **(D)**. MR Leave-one-out sensitivity analysis for *Ruminococcus* 2 on Colorectal Cancer. **(E)**. MR Leave-one-out sensitivity analysis for *Sutterella* on Colorectal Cancer. **(F)**. The funnel plot of *Defluviitaleaceae UCG011* on Colorectal Cancer.).

Cochran’s Q test and the *I* ^*2*^ statistic were conducted to evaluate heterogeneity among instrumental variable estimates derived from individual genetic variants. The results revealed no significant evidence of heterogeneity, as presented in Table 2. This lack of heterogeneity suggests that the MR estimates are more reliable. The ‘leave-one-out’ analysis, where each SNP was systematically removed to assess its impact on the IVW point estimate (Figures 5A–E), indicated that no single SNP significantly influenced the overall result. The funnel plot exhibited no significant asymmetry, indicating minimal publication bias (Figure 5F). Furthermore, MR-Egger regression and the MR-PRESSO global test both indicated no horizontal pleiotropy, as detailed in Table 2. In summary, the combination of minimal heterogeneity, consistent ‘leave-one-out’ results, and the absence of asymmetry enhances confidence in the reliability of the MR estimates and mitigates concerns regarding bias.

**TABLE 2:** Results of heterogeneity and pleiotropy test.

Exposures	Outcome	Heterogeneity test					Pleiotropy test				
		Methods	*Q*	*df*	*Q-val*	*I^2^*	MR egger regression intercept	MR egger standard error	MR egger directionality *p*-value	MR-PRESSO RSSobs	MR-PRESSO *p*-value
*Eubacterium fissicatena group*	CRC	MR Egger	3.854	7	0.796	0.816	0.062	0.039	0.156	8.282	0.619
		IVW	6.380	8	0.605	0.254					
*Anaerofilum*		MR Egger	6.139	9	0.726	0.466	0.017	0.029	0.577	7.922	0.769
		IVW	6.474	10	0.774	0.545					
*Defluviitaleaceae UCG011*		MR Egger	4.172	8	0.841	0.918	−0.019	0.030	0.557	5.637	0.885
		IVW	4.547	9	0.872	0.979					
*Ruminococcus 2*		MR Egger	5.451	13	0.964	1.385	−0.014	0.014	0.356	7.175	0.973
		IVW	6.366	14	0.956	1.199					
*Sutterella*		MR Egger	11.523	10	0.318	0.132	0.0004	0.027	0.988	13.741	0.421
		IVW	11.523	11	0.401	0.045					

Abbreviations: IVW, inverse variance weighted; MR, mendelian randomization; CRC, colorectal cancer; RSSobs, the observed residual sum of squares.

### Hub gene and gene functions

**FIGURE 6:**
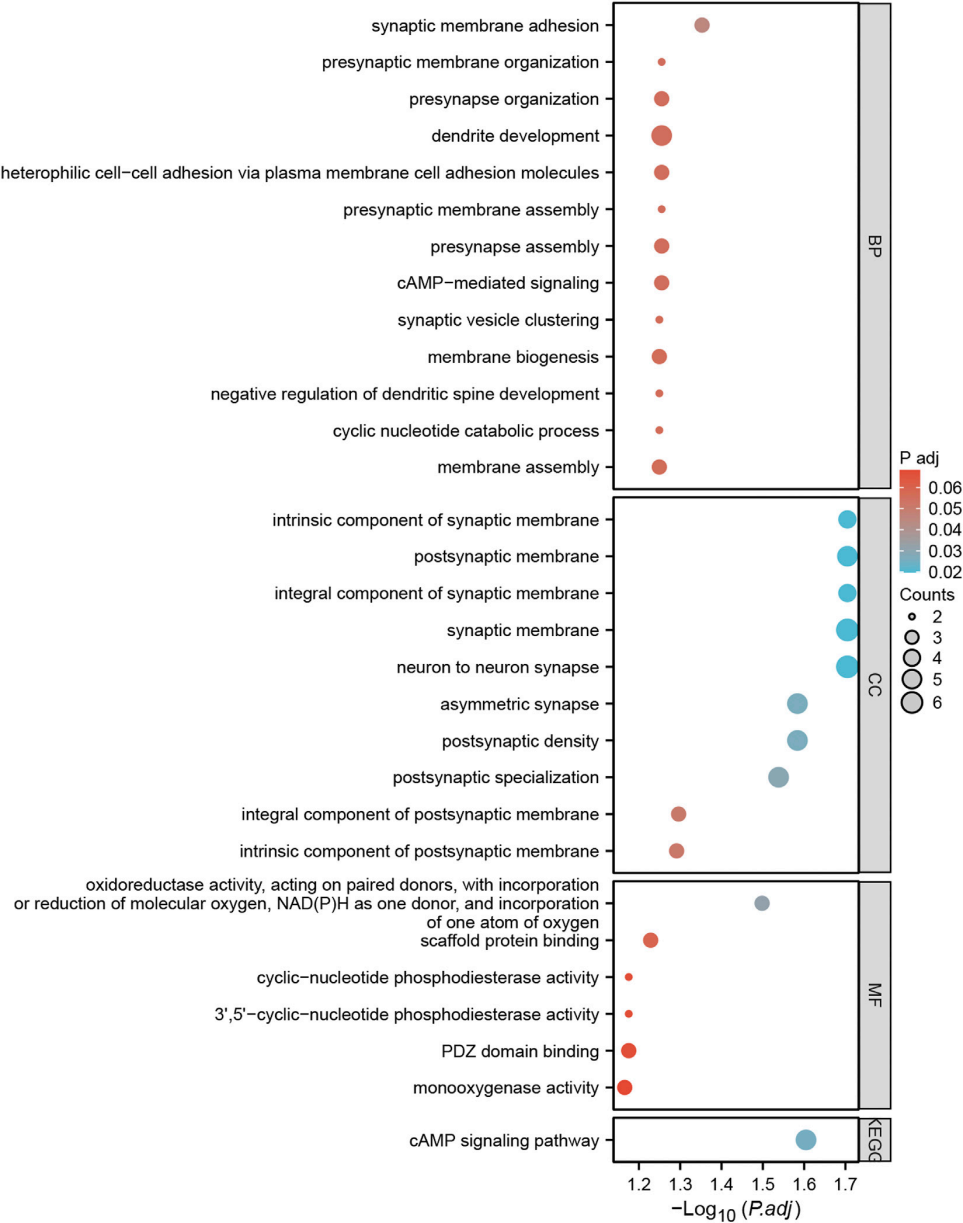
Bubble plots of GO and KEGG pathway enrichment analysis for genes annotated with SNPs.

**FIGURE 7:**
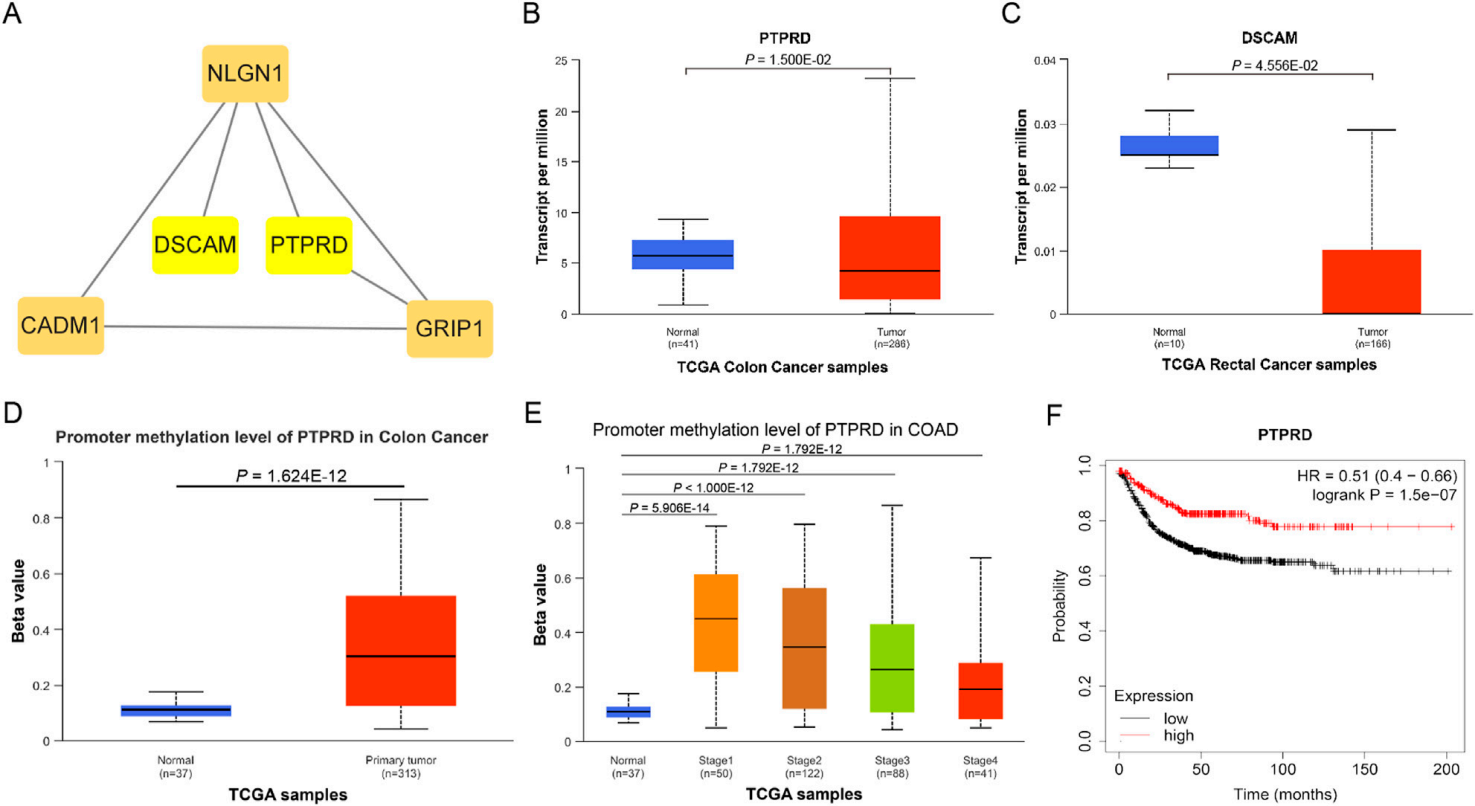
Hub genes and expression in TCGA samples. **(A)**. Hub genes; **(B)**. Expression of *PTPRD* in TCGA Colon cancer samples; **(C)**. Expression of *DSCAM* in TCGA rectal cancer samples; **(D)**. Promoter methylation level of *PTPRD* in TCGA Colon cancer samples (Normal to Tumor); **(E)**. Promoter methylation level of *PTPRD* in TCGA Colon cancer samples (Normal to Different stages); **(F)**. KM survival curve of *PTPRD* in TCGA Colon cancer samples.

A total of 57 SNPs associated with the potential relationship between colorectal cancer and the five microbial genera were included in the MR analysis. Using the R package, we identified all genes within 50 base pairs of these SNPs, resulting in a total of 61 unique genes after removing duplicates. The list of genes is provided in Supplementary Table S4. Pathway analysis through KEGG and GO revealed enrichment in pathways such as the cAMP signaling pathway, synaptic membrane adhesion, cell adhesion, and neuron-to-neuron synapse (Figure 6). Using the STRING online tool and Cytoscape software, we identified five hub genes: *NLGN1, GRIP1, PTPRD, CADM1,* and *DSCAM* (Figure 7A). Among these genes, *PTPRD* and *DSCAM* are located near the SNP sites (rs112893842; rs55658617) analyzed in the *Defluviitaleaceae UCG011* genus MR analysis. Analysis of the TCGA database revealed a significant decrease in *PTPRD* expression in colon cancer, while *DSCAM* showed a significant decrease in rectal cancer (*PTPRD*: *p* = 1.500E-02; *DSCAM*: *p* = 4.556E-02) (Figures 7B,C). Further analysis of the *PTPRD* gene promoter region’s methylation status using TCGA data revealed a significant increase in methylation levels in colon cancer tissues. Moreover, a significant correlation was observed between the methylation levels of the *PTPRD* gene promoter and the staging of colon cancer (methylation levels of the *PTPRD*: *p* = 1.624E-12) (Figures 7D,E). Survival analysis revealed a significant correlation between *PTPRD* gene expression and colon cancer survival time (Figure 7F).

## Discussion

Colorectal cancer is one of the most common malignant tumors of the digestive tract, with incidence and mortality rates rising annually (Bray et al., 2024; Siegel et al., 2022). Recent studies have shown that the gut microbiome plays a complex role in the development and progression of colorectal cancer (Wong and Yu, 2023). Here, we conducted a two-sample Mendelian Randomization analysis and found a potential association between five gut microbial genera and CRC: *E. fissicatena group*, *Anaerofilum*, *Defluviitaleaceae UCG011*, *Ruminococcus 2*, and *Sutterella*. This aligns with findings from previous research (Ni, Li, Zhang, Xu, Wei, Feng, Zhao, Zhang, Zhang, Shen and Li, 2022; Xiang et al., 2023). However, our study identified *Defluviitaleaceae UCG011* as a unique risk factor for CRC, underscoring its potential role in promoting the occurrence and development of colorectal cancer.

*Defluviitaleaceae UCG011* is a genus within the Defluviitaleaceae family, belonging to the Firmicutes phylum, Clostridia class, and Lachnospirales order. Kawamoto et al. observed an increased abundance of *Defluviitaleaceae* in the intestines of patients with periodontitis, suggesting its potential involvement in the body’s inflammatory response (Kawamoto et al., 2021). Another study by Chen et al. found that bacteria from the Defluviitaleaceae family, particularly *Defluviitaleaceae UCG011*, influenced the immune-mediated disease Granulomatosis with Polyangiitis through CD11c in granulocytes, highlighting its role in human immune responses (Chen and Tang, 2023). Additionally, Wang et al. revealed a significant positive correlation between *Defluviitaleaceae UCG011* and susceptibility to the immune-related disease ankylosing spondylitis (Wang et al., 2023). To the best of our knowledge, there is currently no literature reporting the role of *Defluviitaleaceae UCG011* in CRC. Based on our research results, we speculate that the *Defluviitaleaceae UCG011* genus may promote the occurrence and development of colorectal cancer by altering intestinal inflammation and immune responses. However, the specific mechanisms require more in-depth investigation.

To gain a deeper understanding of the link between the five specific microbial genera and colorectal cancer, we conducted a detailed analysis of genes located in close proximity to each SNP identified in our study. Our analysis focused on the results obtained from the MR study. Enrichment analysis revealed that these genes were significantly enriched in the cell signaling pathway and cAMP signaling pathway. Subsequent analysis identified five hub genes: *NLGN1, GRIP1, PTPRD, CADM1,* and *DSCAM*. Among them, the *PTPRD* and *DSCAM* genes are located near the SNP sites (rs112893842, rs55658617) analyzed in the *Defluviitaleaceae UCG011* genus MR analysis. These findings suggest that this microbial genus may play a significant role in biological processes related to colorectal cancer.

*PTPRD* (Protein Tyrosine Phosphatase Receptor Type D) is a protein-coding gene that encodes a protein belonging to the protein tyrosine phosphatase (PTP) family. PTPs are known signaling molecules that regulate various cellular processes, including cell growth, differentiation, the cell cycle, and oncogenic transformation. Relevant pathways include protein-protein interactions at synapses and signal transmission across chemical synapses (Pulido et al., 1995; Veeriah et al., 2009). Through analysis of the TCGA database, we found that *PTPRD* is significantly downregulated in colon cancer tissues, suggesting that *PTPRD* may play a role in inhibiting colon cancer. Further analysis revealed a markedly increased methylation level of *PTPRD* in colon cancer samples, leading us to hypothesize that *PTPRD* inactivation due to methylation may promote the progression of colorectal cancer. *DSCAM* (Down Syndrome Cell Adhesion Molecule) is another protein-coding gene belonging to the immunoglobulin superfamily of cell adhesion molecules (Ig-CAM). It is involved in the development of the human central and peripheral nervous systems (Agarwala et al., 2000) and mediates intracellular signal transduction by activating MAPK8 and P38 MAP kinases (Liu et al., 2009; Ly et al., 2008). We examined the expression of these two genes in colorectal cancer using the TCGA database and found that *PTPRD* expression was significantly decreased in colon cancer, while *DSCAM* expression was significantly decreased in rectal cancer. This may be related to differences in intestinal bacterial diversity, and further investigation is needed to understand the precise mechanisms. Moreover, our analysis of *PTPRD* gene methylation levels using TCGA data revealed a substantial increase in methylation within the *PTPRD* promoter region of tumor tissues. Notably, this elevated methylation status was strongly associated with the stage of CRC. This suggests that *Defluviitaleaceae UCG011* may influence the expression of *PTPRD* by altering methylation levels in its promoter region, thereby contributing to the development and progression of CRC. Survival analysis revealed a significant correlation between *PTPRD* gene expression and colon cancer prognosis, highlighting the potential role of *Defluviitaleaceae UCG011* in modulating the initiation and progression of CRC.

While our research provides valuable insights into the underlying mechanisms involved in the development and progression of CRC, it does have several limitations. 1) The GWAS data used in this research are derived exclusively from European populations, and there is a known disparity in gut microbiota diversity across different populations. Therefore, the generalizability of our findings to other regional population may require further validation. 2) With the rapid evolution of technology, the functionality of genetic variations may change, and the SNPs included in our study may not be suitable for future research. 3) The information provided by the gut microbiota GWAS at the species or strain level is limited, reducing the precision of two-sample MR analyses in accurately inferring potential associations. 4) Despite preliminary MR results suggesting potential connections between gut microbial genera, genes, and CRC, this relationship loses significance after *p*-value correction, possibly due to limited sample size or a small effect size. The associated microbial genera and genes identified in this study require further in-depth research and clinical validation due to existing limitations in this study.

In conclusion, our study identified five gut microbial genera potentially linked to CRC, with only one genus, *Defluviitaleaceae UCG011*, identified as a risk factor for CRC. Through SNP annotation, we pinpointed two hub genes associated with *Defluviitaleaceae UCG011*: *PTPRD* and *DSCAM*. Our analysis suggests that these genes may play a role in developing CRC, and *Defluviitaleaceae UCG011* may potentially influence the development and progression of CRC. Our study offers new insights on researching the mechanisms involved in the onset and progression of CRC. Therefore, we recommend that in-depth investigations be conducted into the role of gut microbial genera in CRC.

## Data Availability

Publicly available datasets were analyzed in this study. This data can be found here: MiBioGen database (https://mibiogen.gcc.rug.nl/) and Finngen database (https://www.finngen.fi/).

## Supplementary material

SUPPLEMENTARY TABLE S1 Details and F-statistics for Each SNP in the Exposure.

SUPPLEMENTARY TABLE S2 MR Analysis Results for all gut Microbial Genera.

SUPPLEMENTARY TABLE S3 FDR of IVW method.

SUPPLEMENTARY TABLE S4 Nearest Gene to SNPs Associated with the Five Microbial Genera Potentially Related to Colorectal Cancer.
